# Maternal Supplementation with LGG Reduces Vaccine-Specific Immune Responses in Infants at High-Risk of Developing Allergic Disease

**DOI:** 10.3389/fimmu.2013.00381

**Published:** 2013-11-26

**Authors:** Paul V. Licciardi, Intan H. Ismail, Anne Balloch, Milton Mui, Edwin Hoe, Karen Lamb, Mimi L. K. Tang

**Affiliations:** ^1^Allergy and Immune Disorders, Murdoch Childrens Research Institute, Melbourne, VIC, Australia; ^2^Department of Paediatrics, University of Melbourne, Melbourne, VIC, Australia; ^3^Clinical Epidemiology and Biostatistics Unit, Murdoch Childrens Research Institute, Melbourne, VIC, Australia; ^4^Allergy and Immunology, Royal Children’s Hospital, Melbourne, VIC, Australia

**Keywords:** vaccine, LGG, probiotic, pneumococcal, Treg, immune modulation

## Abstract

Probiotics are defined as live micro-organisms that when administered in adequate amounts confer a health benefit on the host. Among their pleiotropic effects, inhibition of pathogen colonization at the mucosal surface as well as modulation of immune responses are widely recognized as the principal biological activities of probiotic bacteria. In recent times, the immune effects of probiotics have led to their application as vaccine adjuvants, offering a novel strategy for enhancing the efficacy of current vaccines. Such an approach is particularly relevant in regions where infectious disease burden is greatest and where access to complete vaccination programs is limited. In this study, we report the effects of the probiotic, *Lactobacillus rhamnosus* GG (LGG) on immune responses to tetanus, *Haemophilus influenzae* type b (Hib) and pneumococcal conjugate (PCV7) vaccines in infants. This study was conducted as part of a larger clinical trial assessing the impact of maternal LGG supplementation in preventing the development of atopic eczema in infants at high-risk for developing allergic disease. Maternal LGG supplementation was associated with reduced antibody responses against tetanus, Hib, and pneumococcal serotypes contained in PCV7 (*N* = 31) compared to placebo treatment (*N* = 30) but not total IgG levels. Maternal LGG supplementation was also associated with a trend to increased number of tetanus toxoid-specific T regulatory in the peripheral blood compared to placebo-treated infants. These findings suggest that maternal LGG supplementation may not be beneficial in terms of improving vaccine-specific immunity in infants. Further clinical studies are needed to confirm these findings. As probiotic immune effects can be species/strain specific, our findings do not exclude the potential use of other probiotic bacteria to modulate infant immune responses to vaccines.

## Introduction

The success of vaccination as a public health measure is best illustrated by the substantial reductions in rates of infectious diseases such as smallpox, polio, tetanus, and diphtheria following the introduction of these vaccines in the early part of the nineteenth century (1). Since that time, vaccination remains one of the most cost-effective health care intervention tools, and global vaccination rates are above 80% in most developed countries (2). The efficacy of vaccines is the result of a combination of factors that include the effectiveness of the specific vaccine, the type of adjuvant included in the vaccine and the achievement of vaccine delivery (completion of recommended schedule) which is in turn influenced by cost and feasibility of route of administration. The term adjuvant comes from the latin “*adjuvare*” meaning “to help.” Adjuvants are critical components of vaccines as they help the immune system respond to the vaccine by several proposed mechanisms such as immunomodulation, cytokine regulation as well as depot formation, which allows for sustained release at the site of injection to maintain a continual source of immune stimulation (3, 4).

Despite these achievements, there are a number of significant challenges that remain. In particular, many children (and adults) living in developing countries still die from vaccine-preventable diseases such as tetanus, pneumococcal disease and rotavirus (5). In the last 10 years, international organizations such as the GAVI Alliance have made a profound impact on child health by improving access and delivery of these life-saving vaccines (6). Continued effort is required to reduce the burden of infectious disease in these settings and it is likely that the combination of increased vaccine coverage as well as the development of novel vaccines and adjuvants will be critical in reducing vaccine-preventable disease globally.

The identification of novel vaccine adjuvants have been the subject of intense scientific research over many decades. Particularly important is their activity at the mucosal surface as well as their ability to target both systemic and mucosal immunity, since most infections occur via the mucosal surface (7). Protection by current vaccines typically relies on recommended schedules that consist of multiple dosing over the first 1–2 years of life. In resource-limited settings, this represents significant challenges in terms of cost and delivery. Adjuvants demonstrated to enhance immunological activity may allow for reduced vaccine dosing in these regions and could be given earlier to protect infants against initial colonization by pathogenic bacteria such as the pneumococcus. The mechanisms of action of current human approved adjuvants such as alum are controversial and have several limitations (8). Many candidate adjuvants have been studied, but only very few are approved for human use due to toxicity issues or a lack of immunogenicity. Therefore, novel adjuvants that can overcome these limitations are required. In recent times, the use of probiotic bacteria as immunomodulators has been investigated owing to their pleiotropic biological effects. Probiotics are defined as “live micro-organisms which when administered in adequate amounts, confer a health benefit on the host” (9). The immunomodulatory activities of probiotic bacteria make them potentially useful candidates as novel adjuvants for human vaccines that require further research.

The most well characterized probiotics include those of the *Lactobacillus* or *Bifidobacterium* species (10). These probiotics have been shown to be safe in humans and animals in a number of studies. In particular, the probiotic *Lactobacillus rhamnosus* GG (LGG) is reported to have a number of immune-modulating effects, including cytokine responses, enhancing protective IgG and IgA levels as well upregulating certain immune cell populations (11, 12). Moreover, LGG has been reported in a limited number of studies to enhance certain vaccine-specific responses (13, 14). Maternal intervention has been one approach to investigate the potential beneficial effects of probiotics such as LGG on increasing early-life protection. In this study, we examined the capacity for the probiotic LGG to enhance immune responses in infants that were part of a larger phase II maternal LGG intervention study for the prevention of allergic disease. Responses to the common childhood vaccines tetanus, *Haemophilus influenzae* type b and the pneumococcal conjugate vaccine (PCV7) were measured as well as total IgG levels.

## Materials and Methods

### Study samples

Plasma and peripheral blood mononuclear cells (PBMCs) samples used in this study were collected from infants of mothers (*n* = 250) that were part of a randomized controlled trial of prenatal LGG for the prevention of eczema (Probiotic Eczema Prevention Study registered with Cochrane Skin Group www.nottingham.ac.uk/ongoingskintrials Trial No. 36) (15). This study was approved by both the Royal Children’s Hospital and Mercy Hospital for Women Research Ethics Committees and all participants gave written informed consent. Mothers were randomized to receive 1.8 × 10^10^ colony forming units LGG (American Type Culture Collection 53103; Dicofarm, Italy) each morning from 36 weeks gestation until delivery, or maltodextrin placebo. At 12 months of age, blood samples (5–10 ml) were collected from infants and in this study, the adjuvant effect of LGG was examined in plasma and PBMC samples collected from infants whose mothers were given LGG (*N* = 31) or placebo (*N* = 30). Participants, clinical trial and laboratory staff were blinded to treatment allocations and immunological assays throughout the study.

### Measurement of tetanus and Hib antibody responses following vaccination

Plasma concentrations of IgG antibodies against *H. influenzae* type b polysaccharide were measured by ELISA using a previously published method (16). 96-well microtiter plates were coated with *H. influenzae* type b polysaccharide conjugated to human serum albumin (BEI Resources, Manassas, VA, USA) overnight at 4°C. The standard anti-Hib polysaccharide serum (Lot 1983, FDA, USA), control anti-Hib human reference serum (NIBSC, UK) and infant samples were added to coated ELISA plates and incubated for 2 h. Horseradish peroxidase-conjugated sheep anti-human IgG (Chemicon, Australia) was used as the detection antibody and a 3.3′, 5.5′-tetramethylbenzidine (TMB) substrate solution (KPL, Gaithersburg, MD, USA) was used for detection. Optical densities were read on a microplate reader at 450 nm (reference filter 630 nm) and converted to μg/ml using KC Junior software (Bio-Tek Instruments Inc., USA).

A commercially available ELISA kit (Genzyme Virotech GmbH, Rüsselsheim, Germany) was used to quantitate plasma concentrations of IgG against tetanus toxoid (TT).

### Measurement of pneumococcal antibody response following PCV7

Plasma levels of pneumococcal serotype-specific IgG were measured using a modified WHO-recommended method (17). Briefly, serotype-specific pneumococcal polysaccharides [American Type Culture Collection (ATCC), USA] were diluted in PBS and adsorbed (coated) onto medium-binding plates (Greiner, Germany) at 37°C for 5 h and then stored at 4°C overnight (O/N). Plates were blocked with phosphate-buffered saline containing 10% (v/v) Fetal Calf Serum (PBS/FCS) and incubated at 37°C for 1 h. Plasma and control samples were diluted 1:100 in a double absorption buffer of PBS/FCS containing cell-wall polysaccharide (CPS; 10 μg/ml) and serotype 22F (30 μg/ml) and incubated overnight at 4°C. A standard serum 89-SF (Food and Drug Administration, USA) was pre-absorbed with CPS only. Following washing of ELISA plates with PBS containing 0.05% (v/v) Tween 20 (PBS-T), serial dilutions of the pre-absorbed 89-SF standard, control, and plasma samples were added and incubated at 37°C for 2 h. Plates were washed with PBS-T and a horseradish peroxidase-conjugated sheep anti-human IgG (Chemicon, Australia) was added and incubated at 37°C for 2 h followed by a further wash step with PBS-T. The reaction was developed by incubation with a TMB substrate solution for 9 min and stopped by the addition of 1 M phosphoric acid. Absorbance at 450 nm (630 nm reference filter) was measured using a microplate reader (Bio-Tek, USA). Pneumococcal serotype-specific IgG concentrations for each sample were derived from the 89-SF standard values and expressed in microgram per milliliter using KC Junior software (Bio-Tek Instruments Inc., USA).

### Measurement of total IgG levels in serum

96-well medium-binding microtiter plates (Greiner, Germany) were coated with 50 ng/ml of unlabeled human IgG (Southern Biotechnology, USA) diluted in carbonate-bicarbonate buffer, pH 9.6 overnight at 4°C. Plates were then blocked with 5% (w/v) skim milk powder in PBS-T for 1 h at RT followed by incubation of standards, serum samples, and controls for 2 h at RT. Level of IgG in serum samples were detected using a 1:5000 dilution of a sheep anti-human IgG-HRP reagent for a further 2 h at RT and developed with a TMB substrate solution for 7 min at RT. Reactions were stopped with the addition of 1 M phosphoric acid and absorbance at 450 nm (630 nm reference filter) was measured using a microplate reader (Bio-Tek, USA). The amount of total IgG in serum samples were derived from the standard curve (in-house control) and expressed in microgram per deciliter using KC Junior software (Bio-Tek Instruments Inc., USA).

### Peripheral blood mononuclear cell isolation and culture

Blood samples (5–10 ml) were collected from infants at 12 months of age into preservative-free sodium heparin tubes. PBMCs were separated from heparinized blood by density gradient centrifugation (Ficoll-Paque, Sweden) within 8 h of collection and rate frozen at a concentration of 8–10 × 10^6^ cells/ml. PBMCs were thawed rapidly at 37°C, washed in RPMI-1640 medium (Gibco, Grand Island, NY, USA) and re-suspended at 1 × 10^6^ cells/ml for culture. Thawed PBMC were cultured in AIM-V serum free medium (Gibco, Grand Island, NY, USA) supplemented with 4 × 10^−5^ M of 2-Mercapethanol (2-ME; Gibco, Grand Island, NY, USA) and stimulated with TT (1 μg/ml) or left in medium alone (with 2-ME; unstimulated) for 6 days at 37°C in 5% CO_2_ in air.

### Enumeration of Treg in PBMCs by flow cytometry

After 6 days of culture, PBMCs were centrifuged at 600 g for 10 min at room temperature and supernatants removed. Cell pellets were stained with fluorochrome-conjugated monoclonal antibodies in 50 μl staining volumes. CD3-allophycocyanin (APC), CD4-peridinin chlorophyll protein (PerCP), CD25-phycoerythrin (PE)-Cy7 (BD Bioscience, San Jose, CA, USA) and forkhead box P3 (FoxP3)-PE (e-Bioscience, San Diego, CA, USA) were used to identify CD25^hi^FoxP3^hi^ T cell populations in PBMCs. The proliferative response to TT was evaluated in 6 day cultures using the cell tracking dye Carboxyfluorescein Diacetate Succinimidyl Ester (CFSE; 0.1 μM). Non-proliferating cells were identified as CFSE^hi^ and proliferating cells as CFSE^lo^. Cells were washed once with PBS and incubated with fluorochrome-labeled antibodies or isotype controls in 50 μl staining volumes for 30 min. For intracellular staining, cells were subsequently permeabilized, fixed, and stained with FoxP3-PE antibody or isotype control according to the manufacturer’s instructions (e-Bioscience). Data were acquired on a 4-color LSR II (BD, San Jose, CA, USA) and analyzed with FACSDiva v4.1 software using well-defined gating strategies. A minimum of 100,000 events were acquired in the lymphocyte gate.

### Statistical analysis

The Student’s *t*-test was used to analyze normally distributed continuous data, and Mann–Whitney *U*-test was used for skewed data. Data were presented as either geometric means with 95% confidence intervals (CI), or median with interquartile range (IQR) depending on the distribution. Frequency data was analyzed using the Fisher’s exact test. A *p*-value <0.05 was considered statistically significant. Analyses were performed using GraphPad Prism Version 6 (GraphPad Software Inc., CA, USA). Linear regression models (or logistic regression when the outcome was binary) were used to adjust for potential confounders in Stata (StataCorp LP, USA) including the presence of IgE-associated eczema ever or at 12 months of age, atopy, and yogurt intake, as there were trends toward significance for these variables between the LGG and placebo groups.

## Results

### Study cohort

A summary of the characteristics of study participants is presented in Table 1. No significant differences were found although there were trends toward a higher proportion of infants who were atopic (46.6 vs. 23.3%; *p* = 0.07) and had reported IgE-associated eczema (33.3 vs. 13.3%; *p* = 0.07) in the LGG group compared to placebo (Table 1). No differences were found for any of the other characteristics.

**Table 1:** **Characteristics of study participants**.

Characteristic	LGG (*n* = 30)^a^	Placebo (*n* = 30)
Infant eczema ever	43.3%	40%
Infant eczema 12 months	40%	23.3%
Infant IgE-eczema ever	33.3%*	13.3%
Infant IgE-eczema 12 months	33.3%*	13.3%
Atopy	46.6%**	23.3%
Paternal eczema	22.6%	21.4%
Maternal eczema	45.2%	51.7%
Sibling eczema	86.4%	60.9%
Antibiotics during pregnancy	29%	23.3%
Daily yogurt intake during pregnancy (g/week), median (range)	200 (0–1400)***	600 (0–1400)
Maternal tertiary education	83.3%	72.4%
Household smoker	13.3%	13.8%
Other children present in household	66.7%	58.6%
Infant sex – female	33.3%	38.7%
Gestation (weeks), median (range)	39.5 (35.4–41.5)	39.5 (37.4–41.5)
Birthweight (g), median (range)	3300 (2700–3975)	3550 (2770–5020)
Cesarean delivery	25.8%	32.1%
Duration of breastfeeding in first year (months), median (range)	8.5 (0–12)	9 (6–12)

*^a^ Characteristics unavailable for one infant*.

*p = 0.07; **p = 0.06; ***p = 0.05 between LGG and probiotic groups.

### Maternal LGG supplementation was associated with reduced vaccine-specific but not total IgG responses in infants

**Figure 1:**
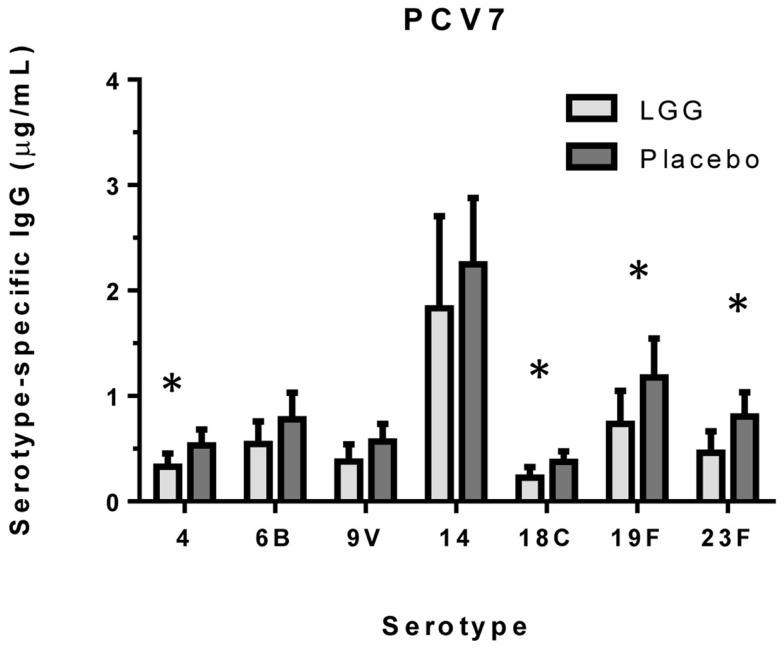
**Serotype-specific IgG levels (GMC ± 95% CI) for infants in the LGG (*N* = 31) or Placebo (*N* = 30) groups following PCV7 immunization**. *p < 0.05 comparing LGG and Placebo treatment.

We examined the impact of maternal LGG supplementation on the antibody response to a number of childhood vaccines in infants at 12 months of age. There were significant differences in the unadjusted GMC for serotype-specific IgG to PCV7 serotypes 4 (*p* = 0.027), 6B (*p* = 0.040), 18C (*p* = 0.032), 19F (*p* = 0.041), and 23F (*p* = 0.019) between the LGG and placebo-treated groups following immunization (Figure 1). Lower serotype-specific IgG levels were observed across all serotypes, with fold decreases of between 0.58 and 0.83 for serotypes 23F and 14, respectively in the LGG group relative to the placebo group. After adjustment for eczema status, a significant difference still remained for each of these serotypes while adjustment for atopy resulted in the serotype-specific IgG response for 9 V also becoming significant (*p* = 0.040; Table A1A in Appendix). Yogurt intake did not affect the PCV7 response after adjustment (Table A1B in Appendix).

**Figure 2:**
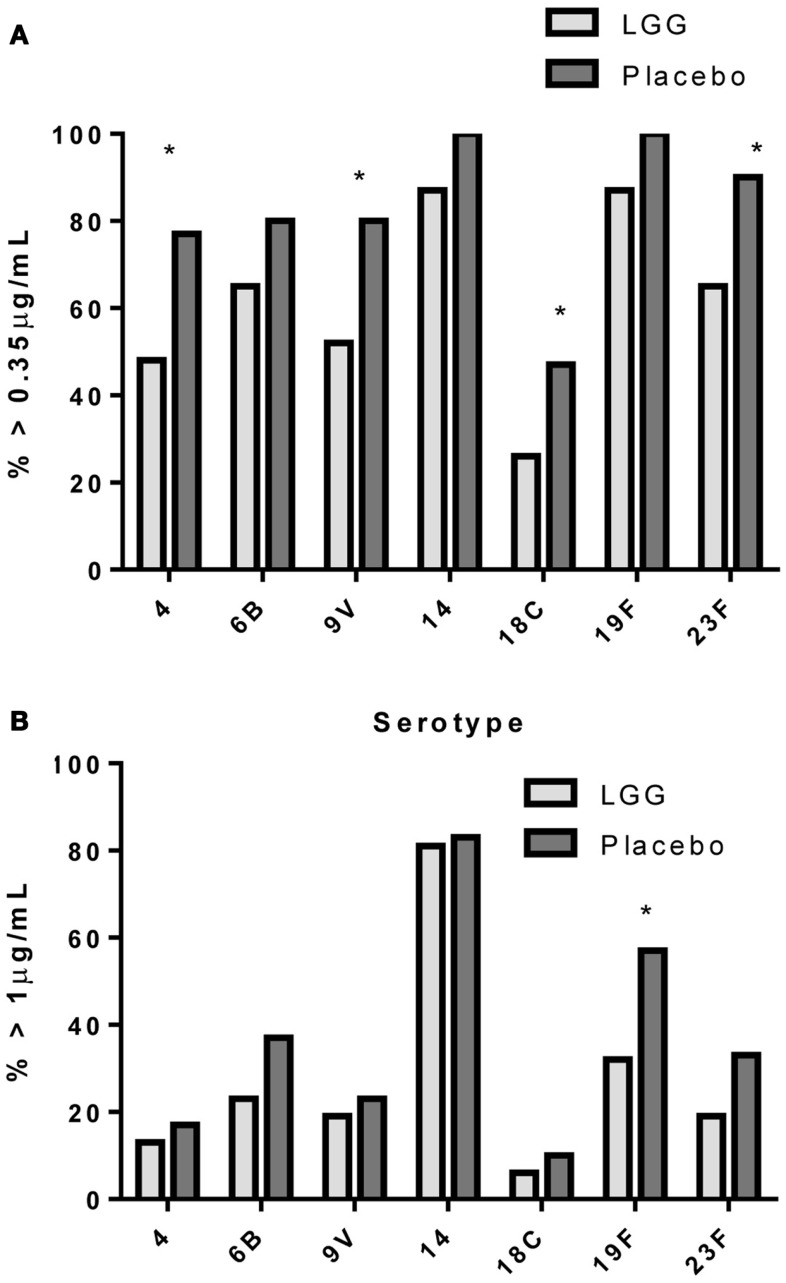
**Proportion of infants with a serotype-specific IgG levels ≥ 0.35 μg/ml (A) or ≥ 1.0 μg/ml (B) for infants in the LGG (*N* = 31) or Placebo (*N* = 30) groups following PCV7 immunization**. *p < 0.05 comparing LGG and Placebo treatment.

In addition, there was a significantly lower proportion of infants with serotype-specific IgG antibody titers >0.35 μg/ml (indicative of a protective response post-PCV7) in the LGG group compared to placebo for four of the seven serotypes in PCV7 (serotypes 4, 9V, 18C, and 23F, all *p* < 0.05; Figure 2A). However, using a cut off of 1.0 μg/ml of serotype-specific IgG, there were no differences between infants of LGG and placebo-treated mothers (although there was a trend for serotype 19F; *p* = 0.058) (Figure 2B). Adjustments for eczema status, atopy, or yogurt intake did not alter the conclusions reached about the impact on this response (Tables A2A,B and A3A,B in Appendix).

**Figure 3:**
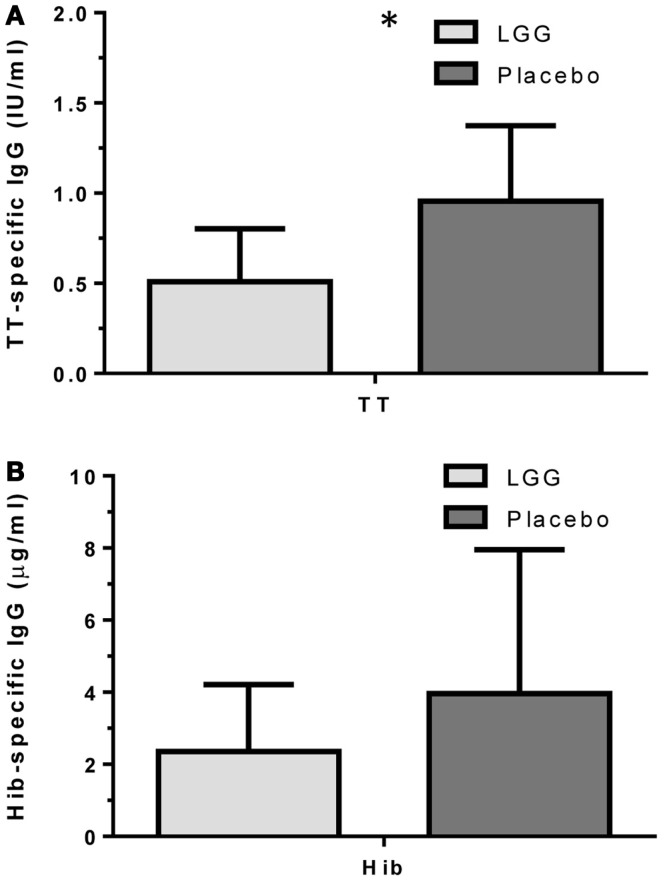
**Levels of (A) anti-TT IgG and (B) anti-Hib IgG (GMC ± 95% CI) for infants in the LGG (*N* = 31) or Placebo (*N* = 30) groups following TT and Hib immunization**. *p < 0.05 comparing LGG and Placebo treatment.

**Figure 4:**
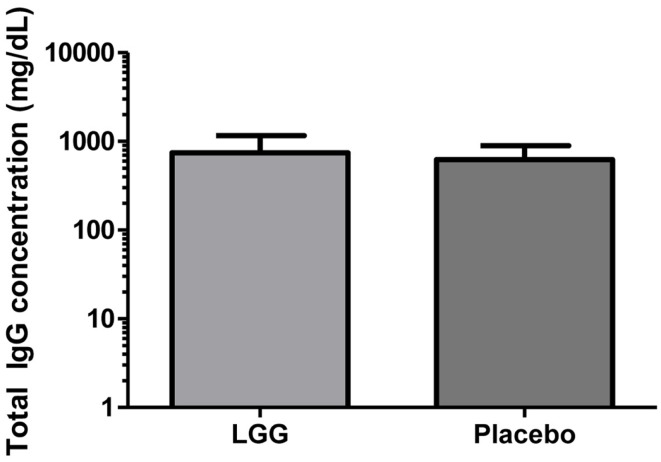
**Level of total IgG in the serum of infants (GMC ± 95% CI) in the LGG (*N* = 31) and Placebo (*N* = 30) groups**.

Plasma levels of anti-tetanus toxoid IgG were also significantly reduced in infants of mothers treated with LGG compared to infants of placebo-treated mothers, with unadjusted GMCs of 0.96 and 0.51 IU/ml (*p* = 0.042) in the LGG and placebo treatment groups respectively, representing a 1.9-fold difference between groups (Figure 3A). After adjustment for each of the potential confounders, the observed association weakened but remained statistically significant (*p* < 0.05) for all variables other than IgE-associated eczema at 12 months of age (*p* = 0.052; Tables A1A,B in Appendix). Similarly, reduced anti-Hib IgG levels were also found in the LGG group compared with placebo, with GMCs of 3.96 and 2.35 μg/ml, respectively, although this was not significant (Figure 3B). Similarly, adjustment for each of the variables weakened this association but did not affect the conclusions about differences between LGG and placebo (Tables A1A,B in Appendix). However, LGG supplementation did not have any effect on total IgG levels in the serum of these infants (Figure 4).

### Maternal LGG supplementation was associated with increased frequency of TT-specific Treg in infants at 12 months of age

**Figure 5:**
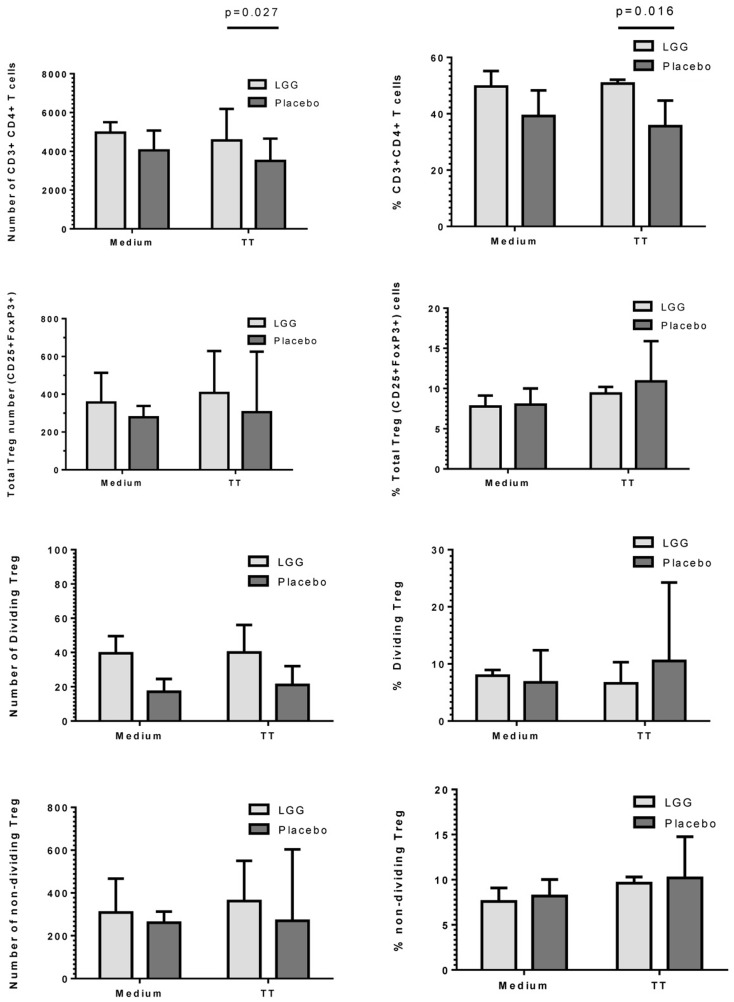
**Numbers and frequency (%) of CD3^+^CD4^+^ T cells, total CD4^+^CD25^+^FoxP3^+^Treg, dividing (CFSE^lo^), and non-dividing (CFSE^hi^) Treg identified in PBMCs isolated from infants in the LGG (*N* = 31) or Placebo (*N* = 30) groups**. PBMCs (1 × 10^6^/ml) were stimulated with TT (1 μg/ml) or unstimulated (medium) for 6 days at 37°C and 5% CO_2_. Bars represent median + interquartile range (IQR). Significance determined using the Mann–Whitney *U*-test.

As LGG has been suggested to promote tolerogenic T regulatory (Treg) responses, we investigated the effect of maternal LGG treatment on Treg responses to the vaccine antigen in infants at 12 months of age. There was a significantly higher number and percentage of TT-specific CD4^+^ T cells in infants of LGG-treated mothers compared to infants of placebo-treated mothers (4563 vs. 3505; *p* = 0.027; 50.7 vs. 35.6%; *p* = 0.016) after stimulation with TT (Figure 5). In addition, there was a non-significant increase in total Treg numbers following stimulation with TT in infants of LGG-treated mothers as compared to infants of placebo-treated mothers (LGG group = 407 vs. placebo group = 305). This was also observed for Treg within both the proliferating CFSE^lo^ population of CD4^+^ T cells (presumed TT-specific Treg; LGG = 40 vs. placebo = 21) as well as the non-dividing CFSE^hi^ population of CD4^+^ T cells (presumed to be naive Treg that lack TCR specificity for TT; LGG = 362 vs. placebo = 270) (Figure 5). Only TT was measured for this response as this part of the panel investigated during the larger PEPS allergy trial as no PBMCs were available for additional analyses.

**Figure 6:**
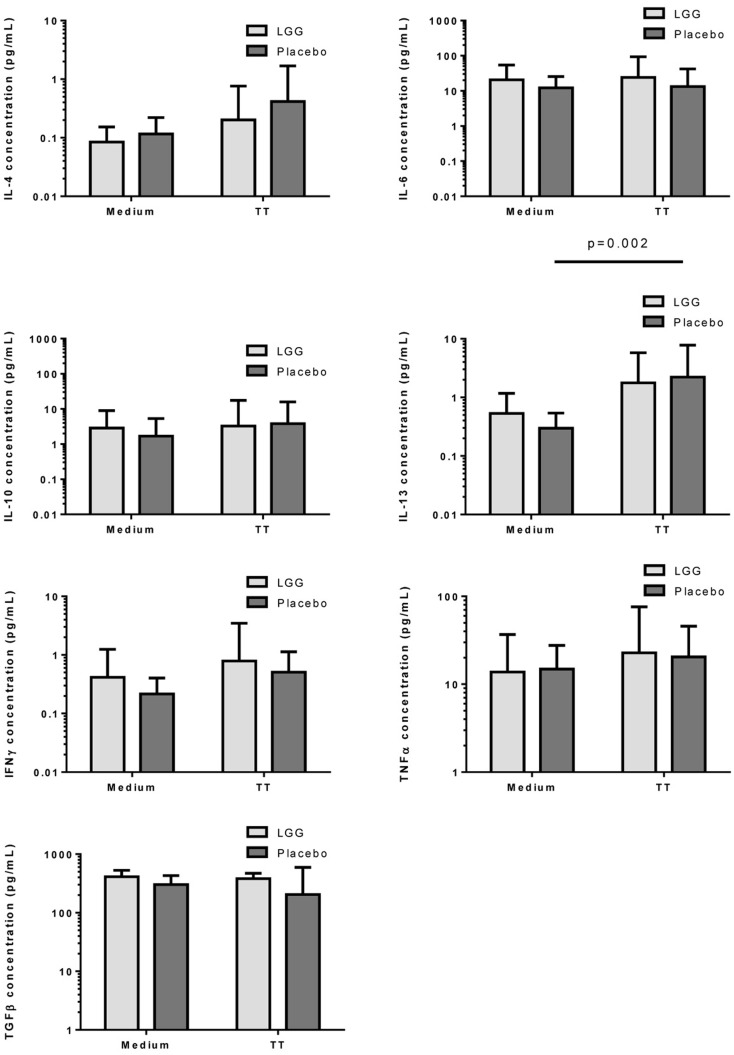
**Levels of IL-4, IL-6, IL-10, IL-13, IFNγ, TNFα, and TGFβ in PBMC supernatants following stimulation with TT (1 μg/ml) or unstimulated (medium) for 6 days at 37°C and 5% CO_2_**. Bars represent GMC ± 95% CI.

Measurement of cytokine responses to TT revealed no significant differences between infants in the LGG and placebo groups for any of the cytokines examined (Figure 6). Interestingly nevertheless, levels of TGF-β produced by PBMCs from infants in the LGG group were noted to be almost two-fold higher than levels of infants in the placebo group (Figure 6).

## Discussion

This study reports that maternal supplementation with the probiotic *L. rhamnosus* GG (LGG) was able to reduce the levels of vaccine-specific antibodies in infants at 12 months of age. It was found that the IgG response to TT and PCV7 vaccines was significantly lower amongst infants of mothers treated with LGG during pregnancy. However no effects on the IgG response to Hib vaccine were observed. Moreover, total IgG levels were similar across the groups, and consistent with previous data (18), suggesting that maternal LGG may be able to modulate, directly or indirectly, specific immune responses in the infants. This is the first study to examine this immune-modulating potential of LGG when administered to mothers during pregnancy and includes a comprehensive evaluation of the immune response to a broad range of vaccine antigens.

In our study, LGG reduced the antibody response to all vaccines studied, with significant reductions observed for TT and four of seven PCV7 serotypes (4, 18C, 19F, and 23F). There was also a reduced proportion of infants with protective IgG levels (≥0.35 μg/ml) to several PCV7 serotypes at 12 months of age. The effect of LGG on the anti-TT response was also associated with increased numbers of TT-specific Treg, although this was not statistically significant. The higher number of Treg – but not percentage of Treg in the CD4^+^ T cell population – is most likely a reflection of the significantly higher number and proportion of CD4^+^ T cells in the LGG group compared to placebo. These data suggest that LGG may induce Th1 responses that are transferred to these allergic infants in early life, consistent with LGG’s reported effects in allergy involving upregulated TT-specific CD4^+^ Treg which may explain the lower TT-specific IgG levels observed in this study. In addition, the elevated (non-significant) TGF-β levels in PBMC supernatants from infants in the LGG group also provide further support for a possible Treg-mediated effect for TT. It is possible that the relatively small sample size in this study may have precluded our ability to detect any real differences by LGG.

The infants in this study were part of a larger randomized, placebo-controlled trial for eczema prevention (termed PEPS) and were considered “high-risk” for the development of allergic disease. Maternal LGG supplementation in this study was not able to prevent the development of eczema in infants by 12 months of age compared to placebo treatment (15).

However, in this study, more infants had reported eczema or atopy in the LGG group compared to placebo and it is possible that the LGG effect on vaccine responses were in part due to their allergic status. In allergy, the immune system is dysregulated with a predominately Th2-biased response characterized by increased levels of IgE and cytokines such as IL-4 and IL-13 while IgG levels and Th1 cytokines such as IFN-γ are reduced (19, 20). A shift toward Th2-based IgE responses in allergy is known to downregulate Th1-based IgG levels – which are typically induced following vaccination – and so it may be possible that the vaccine-specific responses seen in the LGG group were a result of their allergic status rather than the probiotic itself. No differences were observed in relation to the Th1:Th2 balance between these groups despite a trend toward higher levels of the regulatory cytokine TGF-β in the LGG group. However, following adjustment for eczema status and atopy, this effect for LGG persisted, indicating that allergic status did not bias these results. It is therefore likely that the effect of LGG was related to an induction of maternal tolerogenic responses that were transferred to the infant. The fact that maternal LGG did not reduce total IgG levels in the infant provides further support to the ability of LGG to modulate certain antigen-specific responses. The IgG response to an unrelated (non-vaccine) antigen was not undertaken in this study but is expected to be low in this cohort given the age of the infants and the relative lack of exposure to antigens other than those in the vaccines. This is best illustrated for pneumococcal vaccination, whereby infants previously given three doses of PCV7 during infancy produce elevated IgG responses to vaccine serotypes but not non-vaccine serotypes at 12 months of age (21). Furthermore, the use of total IgG as a non-vaccine antigen control for TT-responses has been reported previously (22).

There are several mechanisms by which probiotics mediate their tolerogenic effects which may help to explain our results observed. A number of studies have reported that probiotics such as LGG are able to increase the number and function of allergen-specific Treg in mice (23) and humans (24). This may occur through direct cell-cell contact or via the release of regulatory cytokines such as TGF-β and IL-10 (25–27) and are critical for the control of immune-mediated diseases (28) and have been reported to correlate with the amelioration of clinical allergy (29–31). Maternal supplementation with probiotics has been shown to induce immunomodulatory effects in infants via cord blood, breast milk, or indirectly through changes to intestinal microbiota (32, 33). We recently reported that LGG reduced the levels of TGF-β and total IgA in breast milk (34) which may explain the lack of effect against allergic disease. It is possible that the maternal LGG was transferred to the infants in this study resulting in changes to the intestinal microbiome. It is now known that the microbiome is a critical factor in shaping the infant immune system by providing essential signals that drive healthy immune development (35).

Probiotics have also been increasingly used as a strategy to restore intestinal dysbiosis that is associated with allergic disease. Early-life interactions between intestinal microbiota and the immune system is critical for the development of a healthy immune system (36). Altered microbiota containing fewer beneficial bacteria are suggested to provide inappropriate signals to mucosal immune cells leading to aberrant inflammatory responses and a loss of immune regulation. Studies have reported that prenatal and/or postnatal probiotic treatment with various *Lactobacillus* and *Bifidobacterium* species improved microbiota patterns (37, 38). Moreover, data from meta-analyses on the ability of probiotics to prevent allergic diseases are inconclusive, owing predominately to the heterogeneity in the study designs and probiotic used (39, 40). However, prenatal probiotic treatment has shown the most promise in this context (41), suggesting that early-life signals are important in immune development. It is possible that LGG transfer from mother to infant may explain the immunomodulatory effect for LGG in this study. Indeed, in the PEPS study, maternal supplementation with LGG increased the numbers of the beneficial bacteria *B. longum* (42) which are important for healthy immune development. Investigations are currently ongoing as to whether the presence of *B. longum* influenced the Th1:Th2 balance in these infants at 12 months of age. It is possible that such early-life influences on microbiota may impact on the immune response to vaccines, particularly in the first 6 months of life. Indeed, it is now recognized that the intestinal microbiome should be considered when developing vaccines specific for certain geographical regions and populations since vaccine efficacy may vary based on the microbial composition of the gastrointestinal system (43).

More recently, the use of probiotics as novel vaccine adjuvants has been investigated (44). Infants treated with LGG at 2–5 months of age had an elevated (but not significant) serum IgA and IgM response following an oral rotavirus vaccine, as well as significantly higher numbers of rotavirus-specific IgM secreting cells compared to placebo (13). LGG was also shown to enhance neutralizing antibody titers as well as serum poliovirus-specific IgG and IgA in adults immunized with the trivalent oral polio vaccine (OPV) compared to placebo (45). In another study, a greater proportion of LGG-treated adults had higher numbers of Typhi-specific IgA secreting cells (ASCs) following oral *S. typhi* Ty21a immunization despite no differences in the specific antibody response (46). In addition, probiotics have been shown to elicit adjuvant properties following immunization with other vaccines such as influenza (14, 47, 48), Hep B (49), and polio (50).

The identification of novel adjuvants that can be administered in early-life would provide a substantial benefit in settings of high infectious disease burden. The contribution of the pneumococcus, Hib, and tetanus to neonatal and infant mortality is considerable, with more than two million deaths each year (51, 52). Furthermore, in developing countries, vaccine delivery is a significant problem where access to complete vaccination schedules is limited or where drop-out rates are high (53). Therefore probiotics potentially offer substantial advantages as vaccine adjuvants in terms of safety, ease of administration and their demonstrated ability to enhance immune responses. The reduced proportion of protective antibody levels in the LGG group may pose a theoretical increased risk of disease susceptibility which would be particularly important in high burden of disease settings. However, as this study was not designed to specifically address the effect of probiotics on vaccine responses, further large scale randomized trials are required to fully evaluate this effect.

A major strength of this study is the comprehensive evaluation of LGG’s capacity to modulate infant vaccine responses following maternal supplementation, involving pneumococcal, Hib, and TT-specific immune responses. In addition, this is one of the first studies to describe the adjuvant effect of probiotics for PCV7 using WHO-based assays. However, several limitations need to be addressed. This is a relatively small cohort and so caution must be applied when considering the results of this study. The effect of multiple comparison testing cannot be excluded and in this study was not performed due to the small sample size. Furthermore, this study may not be adequately powered to detect a beneficial impact of LGG on infant vaccine responses given the primary outcome was clinical allergy at 12 months of age. Also, maternal LGG supplementation may not be as effective as a combined prenatal/postnatal or postnatal alone approach for examination of the infant immune response to vaccines as has been shown for studies of allergic disease (10). Moreover, all vaccinations were administered as part of the 3-dose National Immunization Program in Australia which might not be optimal to detect any differences in these responses, as all recipients would be expected to respond robustly to this schedule (54). Examination of earlier post-vaccination time-points may have potentially revealed important differences by LGG. It is also possible that probiotics other than LGG may be more beneficial in this setting since it is known that the activities of probiotics are dependent on the species and strain used (18). Subsequent studies in a larger healthy cohort will be important in understanding the potential benefits of probiotics in modulating vaccine-specific immune responses during early-life.

## Conclusion

The probiotic LGG was found to reduce the antibody levels specific for TT, PCV7, and Hib but not total IgG in infants at 12 months of age who were part of a maternal supplementation trial for the prevention of allergic disease. These results suggest that this probiotic may not be beneficial in relation to improving vaccine-specific responses in these infants. Various factors may have impacted on this response such as the nature of the cohort, timing of probiotic administration, or the probiotic itself. Therefore additional studies that are designed to specifically address these questions will be of significant interest, particularly in settings of high infectious disease burden and where access to complete vaccine schedules is limited.

## Conflict of Interest Statement

The authors declare that the research was conducted in the absence of any commercial or financial relationships that could be construed as a potential conflict of interest.

## Appendix

**Table A1A:** **Summary of the vaccine-specific IgG responses following adjustment for eczema and atopy**.

		Adjusted		
Vaccine	Unadjusted^a^	IgE-eczema (at 12 months)	IgE-eczema (ever)	Atopy
PCV7–4	0.47 (0.21); *p* = 0.027	0.51 (0.21); *p* = 0.019	0.50 (0.21); *p* = 0.022	0.50 (0.21); *p* = 0.023
PCV7–6B	0.43 (0.20); *p* = 0.040	0.48 (0.21); *p* = 0.025	0.50 (0.21); *p* = 0.020	0.45 (0.21); *p* = 0.040
PCV7–9V	0.39 (0.22); *p* = 0.085	0.45 (0.23); *p* = 0.055	0.44 (0.23); *p* = 0.060	0.47 (0.23); *p* = 0.040
PCV7–14	0.19 (0.23); *p* = 0.413	0.23 (0.24); *p* = 0.344	0.24 (0.24); *p* = 0.316	0.23 (0.24); *p* = 0.337
PCV7–18C	0.48 (0.22); *p* = 0.032	0.51 (0.22); *p* = 0.027	0.52 (0.22); *p* = 0.024	0.53 (0.22); *p* = 0.021
PCV7–19F	0.47 (0.22); *p* = 0.041	0.47 (0.23); *p* = 0.047	0.48 (0.23); *p* = 0.045	0.47 (0.23); *p* = 0.047
PCV7–23F	0.54 (0.22); *p* = 0.019	0.57 (0.23); *p* = 0.017	0.58 (0.23); *p* = 0.015	0.54 (0.23); *p* = 0.022
Tetanus toxoid	0.60 (0.289); *p* = 0.042	0.60 (0.30); *p* = 0.052	0.63 (0.30); *p* = 0.039	0.64 (0.30); *p* = 0.037
Hib	0.56 (0.44); *p* = 0.212	0.50 (0.48); *p* = 0.306	0.51 (0.48); *p* = 0.293	0.57 (0.47); *p* = 0.234

*Data represents the regression coefficient and standard errors in brackets. Regression coefficient refers to the group as a predictor for each of the outcome measures where the baseline for comparison is the LGG group (*n* = 31)*.

*For example, considering PCV7–4 (coefficient of 0.47 in the unadjusted analysis) refers that the mean response for PCV7–4 in the control group (Placebo) is 0.49 times higher than the mean response for the LGG group*.

*^a^ Unadjusted analysis after removing missing data for eczema and atopy variables (*N* = 30 LGG; *N* = 30 Placebo). This refers to the analysis between the LGG and placebo groups after removal of vaccine immune responses for the one infant where the eczema/atopy status was not available*.

**Table A1B:** **Summary of the vaccine-specific IgG responses following adjustment for yogurt intake**.

	Yogurt intake	
Vaccine	Unadjusted^a^	Adjusted
PCV7–4	0.66 (0.22); *p* = 0.005	0.56 (0.22); *p* = 0.015
PCV7–6B	0.28 (0.23); *p* = 0.229	0.20 (0.23); *p* = 0.390
PCV7–9V	0.56 (0.24); *p* = 0.025	0.53 (0.25); *p* = 0.041
PCV7–14	0.32 (0.25); *p* = 0.200	0.31 (0.26); *p* = 0.229
PCV7–18C	0.63 (0.24); *p* = 0.011	0.59 (0.24); *p* = 0.019
PCV7–19F	0.56 (0.24); *p* = 0.024	0.52 (0.25); *p* = 0.042
PCV7–23F	0.71 (0.24); *p* = 0.005	0.67 (0.25); *p* = 0.009
Tetanus toxoid	0.64 (0.33); *p* = 0.055	0.68 (0.34); *p* = 0.047
Hib	0.50 (0.46); *p* = 0.286	0.35 (0.48); *p* = 0.463

*Data represents the regression coefficient and standard errors in brackets*.

*^a^ Unadjusted analysis after removing missing data for yogurt intake (*N* = 25 LGG; *N* = 26 Placebo)*.

**Table A2A:** **Summary of the PCV7 serotype-specific IgG responses ***≥***0.35 ***μ***g/ml following adjustment for eczema and atopy**.

		Adjusted		
Vaccine	Unadjusted^a^	IgE-eczema (at 12 months)	IgE-eczema (ever)	Atopy
PCV7–4	3.29 (1.08, 9.95); *p* = 0.035	3.27 (1.05, 10.19); *p* = 0.041	3.30 (1.09, 10.00); *p* = 0.035	3.47 (1.10, 10.96); *p* = 0.034
PCV7–6B	2.32 (0.72, 7.41); *p* = 0.157	3.04 (0.89, 10.35); *p* = 0.075	2.36 (0.73, 7.61); *p* = 0.149	2.43 (0.73, 8.09); *p* = 0.147
PCV7–9V	3.50 (1.11, 11.02); *p* = 0.032	4.07 (1.23, 13.49); *p* = 0.022	3.62 (1.14, 11.51); *p* = 0.030	4.27 (1.26, 14.43); *p* = 0.019
PCV7–14	NA	NA	NA	NA
PCV7–18C	2.41 (0.82, 7.10); *p* = 0.111	2.30 (0.76, 6.96); *p* = 0.142	2.44 (0.82, 7.23); *p* = 0.107	2.93 (0.92, 9.35); *p* = 0.069
PCV7–19F	4.46 (0.47, 42.51); *p* = 0.194	4.96 (0.50, 48.92); *p* = 0.170	4.48 (0.47, 42.76); *p* = 0.192	4.56 (0.46, 45.46); *p* = 0.196
PCV7–23F	5.21 (1.28; 21.24); *p* = 0.021	5.25 (1.25, 22.03); *p* = 0.024	5.21 (1.28, 21.24); *p* = 0.021	4.67 (1.12, 19.55); *p* = 0.035

*Data represents the Odds Ratio and 95% confidence intervals; NA, not assessed due to co-linearity issues*.

*^a^ Unadjusted analysis after removing missing data for eczema and atopy variables (*N* = 30 LGG; *N* = 30 Placebo)*.

**Table A2B:** **Summary of the PCV7 serotype-specific IgG responses ***≥***0.35 ***μ***g/ml following adjustment for yogurt intake**.

	Yogurt intake	
Vaccine	Unadjusted^a^	Adjusted
PCV7–4	4.07 (1.25, 13.24); *p* = 0.020	3.63 (1.09, 12.10); *p* = 0.036
PCV7–6B	1.98 (0.55, 7.16); *p* = 0.300	1.92 (0.51, 7.16); *p* = 0.333
PCV7–9V	5.35 (1.52, 18.75); *p* = 0.009	5.36 (1.48, 19.36); *p* = 0.010
PCV7–14	NA	NA
PCV7–18C	2.71 (0.82, 9.00); *p* = 0.103	2.75 (0.80, 9.39); *p* = 0.107
PCV7–19F	4.76 (0.49, 45.95); *p* = 0.177	3.60 (0.35, 37.09); *p* = 0.281
PCV7–23F	6.02 (1.43, 25.40); *p* = 0.014	5.74 (1.33, 24.79); *p* = 0.019

*Data represents the Odds Ratio and 95% confidence intervals; NA, not assessed due to co-linearity issues*.

*^a^ Unadjusted analysis after removing missing data for yogurt intake (*N* = 25 LGG; *N* = 26 Placebo)*.

**Table A3A:** **Summary of the PCV7 serotype-specific IgG responses ***≥***1.0 ***μ***g/ml following adjustment for eczema and atopy**.

		Adjusted		
Vaccine	Unadjusted^a^	IgE-eczema (at 12 months)	IgE-eczema (ever)	Atopy
PCV7–4	1.30 (0.31, 5.40); *p* = 0.718	1.30 (0.30, 5.64); *p* = 0.726	1.28 (0.31, 5.36); *p* = 0.731	1.50 (0.34, 6.65); *p* = 0.591
PCV7–6B	2.32 (0.72, 7.41); *p* = 0.157	2.21 (0.67, 7.27); *p* = 0.193	2.32 (0.72, 7.41); *p* = 0.157	2.24 (0.68, 7.40); *p* = 0.187
PCV7–9V	1.22 (0.36, 4.17); *p* = 0.754	1.23 (0.35, 4.36); *p* = 0.752	1.20 (0.35, 4.14); *p* = 0.776	1.66 (0.44, 6.25); *p* = 0.456
PCV7–14	1.25 (0.34, 4.64); *p* = 0.739	1.20 (0.31, 4.63); *p* = 0.794	1.24 (0.33, 4.62); *p* = 0.746	1.25 (0.32, 4.82); *p* = 0.751
PCV7–18C	1.56 (0.24, 10.05); *p* = 0.643	NA	NA	NA
PCV7–19F	2.62 (0.92, 7.46); *p* = 0.072	2.32 (0.79, 6.79); *p* = 0.125	2.61 (0.92, 7.45); *p* = 0.073	2.49 (0.85, 7.32); *p* = 0.096
PCV7–23F	2.00 (0.62, 6.46); *p* = 0.247	2.16 (0.64, 7.32); *p* = 0.217	1.98 (0.61, 6.47); *p* = 0.256	2.19 (0.64, 7.43); *p* = 0.210

*Data represents the Odds Ratio and 95% confidence intervals; NA, not assessed due to co-linearity issues*.

*^a^ Unadjusted analysis after removing missing data for eczema and atopy variables (*N* = 30 LGG; *N* = 30 Placebo)*.

**Table A3B:** **Summary of the PCV7 serotype-specific IgG responses ***≥***1.0 ***μ***g/ml following adjustment for yogurt intake**.

	Yogurt intake	
Vaccine	Unadjusted^a^	Adjusted
PCV7–4	2.74 (0.48, 15.65); *p* = 0.257	2.17 (0.35, 13.39); *p* = 0.404
PCV7–6B	1.41 (0.41, 4.86); *p* = 0.589	1.11 (0.30, 4.11); *p* = 0.873
PCV7–9V	1.47 (0.40, 5.45); *p* = 0.561	1.20 (0.31, 4.68); *p* = 0.797
PCV7–14	1.33 (0.35, 5.06); *p* = 0.679	1.33 (0.34, 5.25); *p* = 0.683
PCV7–18C	0.96 (0.12, 7.38); *p* = 0.967	0.80 (0.10, 6.50); *p* = 0.834
PCV7–19F	2.57 (0.80, 8.23); *p* = 0.112	2.20 (0.66, 7.30); *p* = 0.199
PCV7–23F	2.78 (0.73, 10.62); *p* = 0.135	2.31 (0.57, 9.29); *p* = 0.238

*Data represents the Odds Ratio and 95% confidence intervals; NA, not assessed due to co-linearity issues*.

*^a^ Unadjusted analysis after removing missing data for yogurt intake (*N* = 25 LGG; *N* = 26 Placebo)*.
